# Guided Self-Help for Binge Eating Prior to Weight Management: The Experience of Clients and Guides

**DOI:** 10.3390/nu17071103

**Published:** 2025-03-21

**Authors:** Ella Upton, Andrew J. Hill, Gemma D. Traviss-Turner

**Affiliations:** Leeds Institute of Health Sciences, School of Medicine, University of Leeds, Leeds LS2 9LJ, UKg.traviss@leeds.ac.uk (G.D.T.-T.)

**Keywords:** binge eating, weight management, obesity, qualitative research, behaviour change, guided self-help

## Abstract

**Background**: Binge eating disorder (BED) is the most common eating disorder. It is strongly associated with obesity and presents a barrier to effective weight management. This study examined clients’ and Guides’ experiences of a guided self-help (GSH) intervention for adults with binge eating and obesity, delivered prior to weight management. **Methods**: Participants were recruited through a behavioural weight management programme and were offered GSH prior to starting. Nine clients with binge eating and four Guides who supported clients were interviewed about their experiences of receiving or facilitating GSH, using an adapted version of the Client Change Interview (CCI)—a semi-structured schedule reflecting on helpful/unhelpful aspects and of the intervention and attributions of change. Interviews were transcribed and analysed using reflexive thematic analysis. **Results**: Themes were organized under 3 main headings. First, GSH offered something new for both parties and was positively received. Clients were unlikely to have had the space to talk about binge eating before and Guides were positive about offering one-to-one support. Second, participants spoke about a range of positive changes to binge eating and how clients felt about themselves and their social relationships. Third, there was agreement on the importance of clients’ relationship with their Guide, the intervention materials, and a helpful mindset as factors facilitating change. Barriers were some aspects of the intervention and the complexity of clients’ lives. **Conclusions**: Offering GSH targeting binge eating prior to weight management was well received. Some tailoring of the approach is recommended, specifically in terms of training for Guides to help in early client engagement. Further research should determine whether the opportunity of GSH for those with binge eating improves the acceptability and effectiveness of later-offered weight management, and the best sequencing of interventions.

## 1. Introduction

Binge Eating Disorder (BED) is currently the most common eating disorder, affecting around 0.3% adult men and 1.5% adult women worldwide [1,2]. It is characterised by a loss of control over the consumption of large amounts of food, eating alone and/or quickly (and not necessarily when hungry), and distressing emotional experiences such as disgust, guilt, and embarrassment [3]. Even more people with binge eating difficulties who do not meet the criteria for a BED diagnosis struggle with many of the same behavioural and emotional features [4]. Accordingly, the emotion regulation model argues that binge eating occurs in response to avoid challenging emotions, leading to short-term relief but longer-term problems [4]. Whilst eating in response to emotions is a common and not inherently problematic behaviour, using food to regulate difficult emotions may result in a toxic cycle of eating to feel better, then feelings of shame and guilt associated with binge eating. Together, they reinforce the likelihood of further binge episodes and eventual weight gain and present a significant barrier to treatment seeking behaviour [5].

Almost 60% of the population globally are living with overweight (43%) or obesity (16%) [6] and rates are higher in economically developed countries such as the United Kingdom [7]. It follows that many people who binge eat also have lived experience of overweight or obesity [8], which is likely associated with the lack of compensatory behaviours that are the core of BED. Obesity places people at risk of several co-morbidities, including certain cancers, cardio-vascular problems, diabetes, pain, depression, poor self-esteem, and reduced health-related quality of life [9]. Those living in larger bodies also experience frequent mistreatment and social stigmatisation, which may serve to predispose/perpetuate some of the aforementioned difficulties [10]. BED may further exacerbate these difficulties [11].

People living with obesity and binge eating are more likely to seek support for weight loss than for their eating difficulties. An estimated 30% of people seeking weight management have binge eating presentations [12]. Binge eating presents a significant barrier to effective engagement and success (measured by weight loss) in both lifestyle modification and weight management interventions [13,14,15]. Moreover, self-reported binge eating behaviours are associated with greater weight gain over a 2-year period following a weight loss intervention [16].

Alongside being more likely to seek support for weight loss, those with binge eating difficulties are less likely to have been offered treatment for BED. Austin et al.’s [17] review across seven countries found that the average duration of untreated BED was greater than five and a half years (67.4 months), more than twice the average duration of untreated anorexia nervosa (29.9 months). There are several reasons why those with binge eating difficulties may not be receiving support. There is a lack of awareness of BED by both healthcare providers and patients [4]. Weight stigma in the healthcare system is important as this has consistently been shown to negatively impact the quality of care received by those living with obesity [18]. In addition, there are restrictions by specialist eating disorder services which may not be commissioned for people living with obesity (in the UK at least).

Guided self-help (GSH) interventions are the first-line recommended treatment for BED [19]. They are recommended by NICE as having moderate quality of evidence, and better evidenced in group settings than used individually. They represent the first intervention within a stepped-care model of treatment with the recommendation of an assessment at 4 weeks of treatment prior to considering a more intensive intervention such as CBT-ED. No other treatments are recommended for binge eating disorder. In a systematic review of 30 randomized controlled trials we found that GSH was superior to active control interventions and waiting list in improving eating disorder psychopathology and binge eating abstinence [18]. GSH shows a similar pattern of results to both CBT and Interpersonal psychotherapy, in that it achieves a sustained effect on binge eating longer-term, but does not produce weight loss alone [8]. However, unlike traditional therapy approaches, the patient-led nature of GSH has shown that symptom reduction continues to improve over-time [20].

GSH often follows cognitive behaviour therapy principles, offering a self-help resource (e.g., a manual) alongside a limited number of individual sessions with a Guide [21]. Clients are required to complete between-session tasks. Much of this is psychoeducation and presented alongside activities that promote understanding and recovery. Importantly, clients must understand they are integral to change and hold the responsibility to address their difficulties [22,23]. GSH is therefore both client-led and Guide supported. Broadening the provision of GSH outside of mainstream eating disorder services has recently been recommended by experts in the field [24]. Weight management may be one of these services. However, there is very little research looking at the acceptability and effectiveness of GSH within weight management.

To address this gap in understanding, the present study uses ‘Working to Overcome Eating Difficulties’ (WOED) as the GSH intervention. This has been shown to be effective in adults with disordered eating in a randomized control trial [23,25]. It is distinctive in that in addition to a manualized self-help resource and access to Guide support, the intervention includes mandatory training for the Guides. Only those trained in intervention use can act as Guides. The intervention uses a physical manual comprising 7 sections. The content is based on cognitive-behavioural principals and is offered with seven one-hour guidance sessions with a trained Guide over 12 weeks (commencing weekly then tapering off in frequency. For more information on the intervention content see our previous papers [25,26]).

When used in weight management setting specifically, we have shown the intervention to have short-term effectiveness in a sample of 24 participants with obesity and binge eating difficulties attending a community weight management service in the UK. The Guides were community dieticians. Eating disorder psychopathology, internalisation symptoms, control over eating all significantly improved [27]. Reductions in binge eating frequency were apparent but did not reach statistical significance. Psychotherapeutic alliance measures yielded consistently high responses, indicating the importance of positive relationships between Guides and their clients [27]. Furthering this work, we have preliminary evidence of the utility of GSH as a precursor to weight management for clients with binge eating [28]. We found a reduction/cessation of binge eating in 11/13 participants who completed the intervention.

While these preliminary findings are important, we know little of the experiences of those who are central to the intervention: clients and Guides. To make the interventions most effective for future use, we need their perspectives on what components of the intervention were beneficial, or what the barriers were within a weight management setting. The aims of the present study were threefold; firstly, to develop a better understanding of clients’ and Guides’ experiences of facilitating/receiving the WOED GSH intervention in a weight management setting. Secondly, to identify what changes were made and thirdly, to explore the main facilitators and barriers to change while individuals engaged in the intervention. We hypothesized that Guides would be seen as central to guided self-help, by both clients and Guides themselves. We also hypothesized that concerns about weight management would persist, given the context in which the intervention was offered.

## 2. Methods

### 2.1. Study Design

This study was a qualitative interview study with a sample of clients and Guides who received/delivered the GSH intervention for binge eating.

### 2.2. Participants and Recruitment

The WOED GSH intervention was offered to clients living with obesity and binge eating difficulties who had been referred to receive the MoreLife (a UK-based provider) weight management intervention within the UK NHS. Clients were screened for eligibility using the Binge Eating Scale [29] and were offered the GSH intervention if they scored >27 indicating severe binge eating, were 18 years or over, and had a BMI of >35 with comorbidities (or >40 without). Clients were excluded if they were receiving concurrent treatment for an eating/weight problem, had previous binge-purge behaviour, bariatric surgery, were pregnant, experiencing a severe psychiatric or medical condition, or had insufficient English language to engage with the intervention materials. All clients who completed the GSH intervention between March and September 2023, were invited to take part in an interview. Guides were required to have supported at least one client to completion. Contact was made by the clinical manager of MoreLife via email with a participant information sheet and a link to an online consent to be contacted form. No-one declined to be interviewed, however one client was deemed inappropriate as they had been referred for further mental health support and one client did not respond to the consent to be contacted email. Both parties completed online consent forms and were contacted by the primary researcher to arrange interviews at a suitable time. 

### 2.3. Materials and Procedure

**Screening:** Clients completed the **Binge Eating Scale (BES)** [29] to assess eligibility for the GSH intervention. The BES is a 16-domain self-report measure designed to assess the severity of binge eating difficulties. It covers eight behavioural and eight emotional/cognitive components typical of binge eating presentations. The measure has shown good internal consistency and test-retest reliability [30]. The cut score is 17 with scores between 18–26 indicating binge eating as a mild to moderate problem and 27+ indicative of severe binge eating [31].

**Interviews:** Interviews were conducted remotely via zoom/telephone by the lead author (EU) and so were in line with the mode of delivery of both the GSH and MoreLife interventions. At the start of the interview, there was a conversation about the privacy of the interviewee and researcher, a contingency plan for loss of signal/contact, and a statement of confidentiality was read and agreed to. Participants were reminded they had the right to withdraw at any time. The interview schedule was an adapted version of the Client Change Interview (CCI v.5; [32]). The CCI is a semi-structured interview comprised of open-ended questions, purposefully designed to support interviewees to reflect on their experiences of a psychotherapeutic intervention and to find their own words to describe change. The interview schedule focuses on the process of change—asking participants whether changes were made, what they found helpful/unhelpful during therapy, why they think the changes occurred and whether they were expected/unexpected [32]. Modifications largely pertained to the Guide interview schedule, due to the nature of the CCI focusing on individuals who have participated in rather than facilitated a therapeutic intervention. Interviews lasted 25–60 min. At the end of the interview, clients were thanked and given a £10 shopping voucher for their time. Clients were signposted to further support where needed (e.g., BEAT, the UK eating disorder charity website/phone line). A MoreLife clinician was always on-call during every interview in case any distress arose. All clients completed the full interview and there were no withdrawals, however one client became distressed during the interview and one reported a deterioration in mood. Both incidents were reported back to MoreLife. Interviews were recorded, held securely, transcribed, and anonymized. Identifying information was held separately to the transcripts.

**Quantitative data**: Routinely collected quantitative data from MoreLife were obtained to add context to the qualitative findings. These included weight, BMI, gender, age, ethnicity, co-morbidities and index of multiple deprivation (IMD). In addition, we obtained pre- and post- intervention scores for the Binge Eating Scale (BES) [26], Generalised Anxiety Disorder-7 Scale (GAD-7) [33], the Short Warwick-Edinburgh Mental-Wellbeing Scale (SWEMWBS) [34], and the Patient Health Questionnaire-9 scale (PHQ-9) [35].

### 2.4. Ethical Approval

The study was conducted in accordance with the Declaration of Helsinki, and ethical approval was granted prior to study commencement from the NHS Cambridge East Research Ethics Committee on 6 February 2023 (REC 23/EE/0053, IRAS ID 319705). A Data Sharing Agreement was drawn up for the secure transfer of routinely collected data between MoreLife and the University of Leeds (28 November 2022).

### 2.5. Analysis

Transcripts were managed in NVivo and analysed using Braun and Clarke’s reflexive thematic analysis (RTA) [36], from a critical realist epistemology. RTA is similar to the traditional 6-stage framework of thematic analysis [37] (familiarization, coding, initial theme generation, theme development and review, refining and defining, and writing-up), but the researcher is positioned as openly central to the generation of themes rather than separate. Accordingly, a reflexive log was kept and used throughout the Results. The lead author (EU) completed the initial theme generation and then the research supervisors (GTT & AH) supported the development and refining of themes as part of an iterative process. Credibility checks were conducted with one client and Guide.

Demographic and quantitative outcome data routinely collected by MoreLife were analysed using descriptive statistics and presented in tabular form to add context to the qualitative data.

## 3. Results

In total, thirteen interviews were conducted with nine clients and four Guides. Guide participants (pseudonyms G-Charlie, G-Bobby, G-Ash and G-Alex) were a mix of genders and ages, all working as weight management practitioners for MoreLife. None were from a psychological therapy background. G-Charlie was the most experienced guide; all others were new to the role and had only supported 1 or 2 clients through WOED. 

Table 1 presents clients’ (pseudonymized) characteristics and outcome measure results, all provided by MoreLife.

Table 1 indicates that client participants were predominantly female (78%) and White British (78%), with a range of ages. In addition, almost half were managing health conditions in addition to their overweight. This is likely be higher as some disclosed managing problems including anxiety, depression, long COVID, and multiple sclerosis during the interviews. All clients were living with significant obesity. 

In terms of questionnaire assessments, prior to GSH, all clients scored ‘severe’ on the BES. Whilst all raw scores reduced over the intervention, five (55.6%) remained ‘severe’. One was ‘mild-moderate’ and three were no longer demonstrating clinically significant levels of binge eating. All clients started the intervention as moderately to severely depressed on the PHQ-9. For three clients (33.3%) these levels remained consistent pre- and post-GSH and for the other six (66.6%), scores reduced by one clinical category. Clients ranged from not anxious to severely anxious on the GAD-7. For five clients (55.6%) these levels remained consistent pre- and post-GSH. For the other four, scores reduced by one clinical category.

In addition, clients presented as low or average in their mental wellbeing, as measured by the SWEMWBS. For most, these categories remained consistent post-GSH, with some increase in raw scores (indicative of increased wellbeing). 

### 3.1. Clients’ and Guides’ Experiences of Receiving/Facilitating GSH

**Figure 1:**
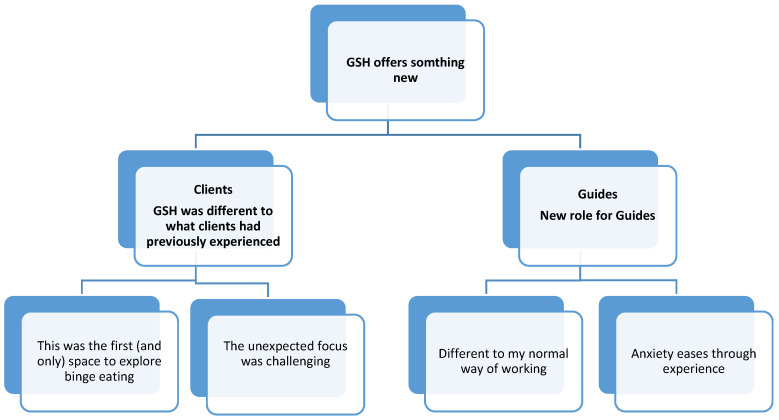
Clients’ and Guides’ experiences, themes and sub-themes.

Clients emphasised the lack of opportunity to discuss binge eating in professional and personal settings, even after actively seeking support from GPs/charities (Figure 1). Contrastingly, GSH provided a space to explore ‘why’ they struggled with weight and BE (rather than focusing on weight loss), in a holistic, person-centered, and reflective way through one-to-one conversations with a non-judgmental other:

‘*I’ve been doing… all the all of them [weight management interventions such as weight watchers/slimming world], probably all different things since I was probably 18, maybe a little bit older, and they’re all just basically the same but slightly different, and no one has ever said to me, there’s got to be a reason, you’re not just greedy, you’re not just unhealthy…*’

(C-Ela)

For many this was the first (and only) space they had to explore binge eating, and therefore, GSH was largely felt to be useful. At times it felt challenging—“*Some of the weeks I found quite hard going emotionally*” (C-Ela). A psychological intervention focusing on the ‘why’ was potentially “*unexpected*” (C-Joy), particularly for clients referred for a weight management intervention. Some commenced GSH without knowing that they had binge eating problems. They had a clear focus on losing weight but hadn’t anticipated this intervention would involve unpicking their relationship with food from a psychological perspective. 

The content and model delivery of GSH was new to Guides too and this, “*Was quite daunting to start off with*” (G-Ash). Guides were used to offering group weight management interventions and some were understandably apprehensive about the shift in role to 1:1 psychological support which was “*different to their normal way of working*” (G-Charlie). Mirroring this inexperience, Guides reflected that the external training was ‘*confusing*’ (G-Alex) and may not have been pitched at the right level for this group of guides. 

However, Guides described enjoying the opportunity to get to know their clients better 1:1 and their initial concerns appeared to be settling through experience. Most of them had only supported around two people through GSH, so it was still very new to them at the time of interview. 

### 3.2. What Were the Changes Made?

**Figure 2:**
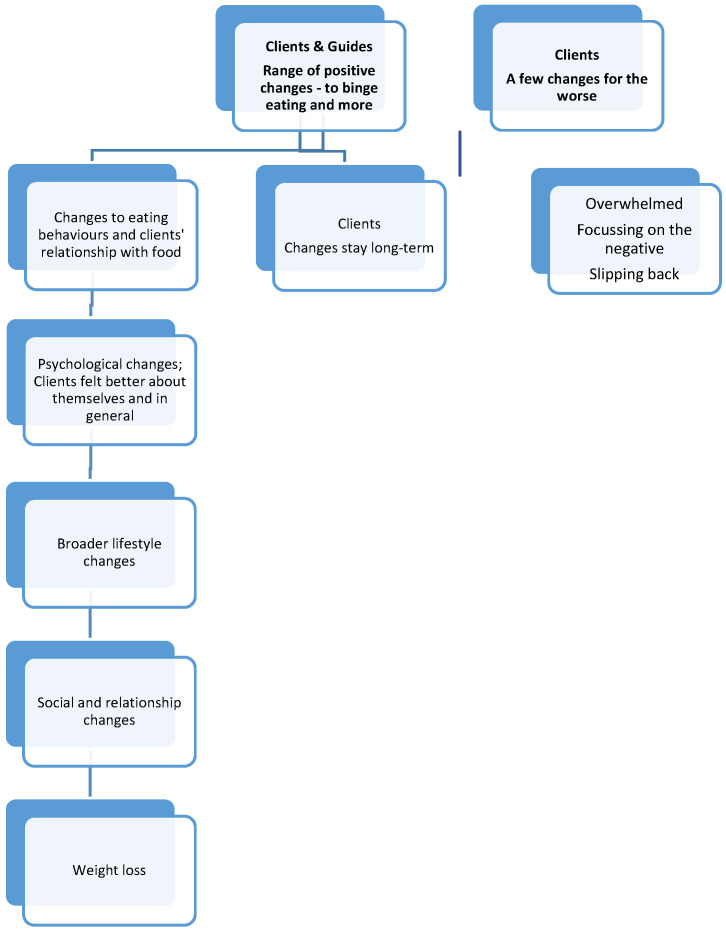
Changes as a result of GSH, organizing themes and sub-themes.

Clients and guides identified a wide range of likely interrelated positive changes associated with GSH (Figure 2). Positive changes in clients’ eating behaviours and associated problem-solving abilities included a reduction in binge eating, having a more balanced/healthy diet, experimenting more with new foods, eating in moderation, eating regular meals, not eating for the sake of eating, not eating unless hungry, not hiding food, and not restricting food. In addition, pausing before eating to think about how it may make them feel, reduced appetite and cravings for highly calorific foods, not shopping when hungry, not keeping food in the bedroom, using distraction instead of eating, and improvements in their ability to deal with relapses in their BE. “*I’m much, much better at just drawing a line underneath. When I know I’ve behaved inappropriately around food I can just draw a line under it.*” (C-Joy)

Other related changes included clients feeling better about themselves and in general (e.g., decreased perfectionism and shame, increased sense of control), “*I’m feeling better because I am in control of it* [binge eating] *and I’m not feeling those guilt feelings and those shame feelings…*” (C-Ela). Similarly, feeling more able to elicit positive social support, “*Now I feel like I’ve got more of a support network*” (C-Tia). There were observations about making behavioural changes such as better bedtime routines and increased exercise, “*I’m more inclined to get up and move around, like physically and mentally*” (C-Ela). In addition, being able to challenge some of their other difficulties (e.g., through getting a volunteering job to combat feelings of loneliness). These positive changes appeared to be linked by an increase in confidence and self-efficacy. Weight loss was also a possible positive change but this was less conclusive.

Two clients who were interviewed several weeks after GSH ended identified that their positive changes had stayed, “*It’s made me feel more aware of like what I can change in myself and it made me feel I have a real good positivity in my life*” (C-Mol).

A minority of clients reported a few changes for the worse. These included feeling overwhelmed, focusing on what they had not achieved, and feeling like they were slipping backwards, “*I hate myself a bit more… because I was doing so well and I’ve just slipped back*” (C-Ray). These difficulties may be compounded by co-occurring difficulties including anxiety and depression, lack of social support, and the ending of the intervention and Guide contact and support. 

### 3.3. Facilitators and Barriers to Change

**Figure 3:**
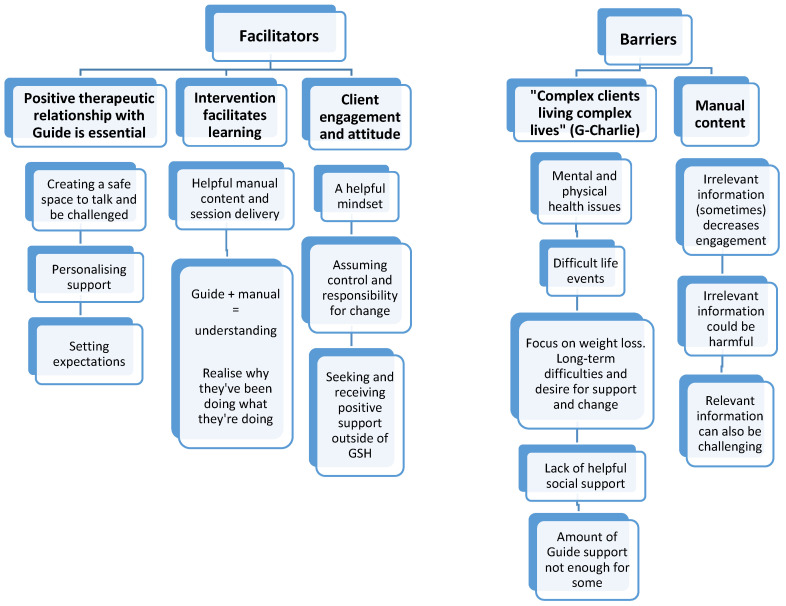
Facilitators and barriers to change, organizing themes and sub-themes.

Guides created a safe space to talk through being “*understanding*”, “*good at listening*” (C-Sue), “*helpful*” (C-Mol), “*sensitive*” (C-Ray), and “*you didn’t feel judged*” (C-Ant; Figure 3). Guides were flexible to client need, whilst setting clear expectations (weight loss is not a goal, the intervention is transdiagnostic so not all information will be relevant, GSH is a client-led intervention), challenging clients and supporting them to problem solve. Without the guide, “*I don’t think it would have been looked at. I think it would have just had a coffee cup stuck on the top of it*” (C-Mol). 

In terms of GSH itself, the content of a psychologically informed accessible manual “*My Bible*” (C-Sue) and sessions with a supportive guide enabled clients to better understand their difficulties with food, eating and previous attempts at weight loss. Messages such as, “*There’s no right and wrong, there’s no food you shouldn’t eat*” (C-Joy) were particularly impactful. This increased understanding was considered instrumental in all the positive changes outlined in Figure 2:

“*What caused these changes?…Becoming aware. Becoming self-aware and reflecting. And getting to the root causes of why I’m me, why I am, who I am, you know, why I am like I am… And then acceptance. And you know, that’s something that perhaps I’ve never ever done before*”.

(C-Joy)

In addition, clients felt that those with positive attitudes towards GSH were more likely to benefit. Having a helpful mindset (e.g., “*I will do my best*” C-Sue), assuming control and taking responsibility for change (“*You’ve got to be able to do things differently*” C-Joy), and actively seeking and receiving support outside of GSH (friends/family/colleagues, mindfulness classes, therapy), were key indicators of fully engaged clients.

In terms of barriers, clients and guides described client-specific difficulties that may impact engagement and success in the short-term, brief and client-led nature of GSH. These included mental health difficulties (e.g., anxiety and depression and their associated symptoms of low motivation and forgetfulness) and physical health concerns (e.g., menopause, pain, stomach problems). “*A couple of them, they did drop out for this reason was because of like stomach issues or medication that was preventing them from establishing a healthy eating routine*” (G-Charlie). Secondly, current or recent external pressures or life events (e.g., financial difficulties, bereavements), “*Having to walk away from a job it’s been hard, so that’s why I probably slipped back*” (C-Ray). Thirdly, a pervasive/stuck focus on weight loss, “*I’d explain quite a bit that you know this has nothing to do with… weight loss or trying to lose weight but quite often the client would, could kind of transfix on that*” (G-Ash). Fourthly, a lack of helpful social support (perhaps perpetuated by the impact of weight stigma). “*I know some of the clients have found it quite difficult talking about this particular topic, it’s very sensitive, they’ve buried it for many, many years, or maybe never spoken about it, so don’t feel comfortable talking about it, and I think a lot of that has to do with the stigma around it…*” (G-Alex). 

Intervention-specific barriers included some of the manual content. For people with binge eating and a lived experience of obesity, having information about what other eating disorder presentations associated with very low weight do (e.g., compensatory behaviours) was not seen as relevant. Importantly, this was seen as potentially impacting engagement, “*I think it kind of acts as a as a catalyst to not to not put as much effort into the rest of it*” (G-Ash). For others, such information felt possibly harmful, “*I had to find a way to staple those pages not to do any of those unhealthy habits* [e.g., purging]” (C-Ant). Other clients were clear that whilst some information was not relevant to them they understood that the GSH content was designed to be delivered transdiagnostically (i.e., regarding all eating disorder presentations) so they were not concerned. Finally, completing food diaries was tricky for some, particularly those embarrassed to share what they were eating. 

## 4. Discussion

This is the first study to investigate the experiences of clients and Guides in receiving/facilitating a GSH intervention addressing binge eating prior to weight management. One of the main themes was that GSH offered something new, with clients and Guides being very positive about this style of support. In terms of positive changes made, these included reductions in binge eating, improvements in psychological wellbeing, and wider behavioural/lifestyle changes. All were likely underpinned by an increase in self-efficacy. As hypothesized, these positive changes were facilitated by the therapeutic relationship between client and Guide, and client engagement with the intervention. In terms of barriers to change, these included clients’ busy lives, their own mental health issues, a lack of social support and, as hypothesized, clients’ persistent focus on weight loss. Further training for Guides in helping people negotiate less pertinent parts of the manual was also highlighted.

One of the novel aspects of this work was that the weight management service provider offered this GSH intervention addressing binge eating prior to clients starting weight management. None of these clients had ever received support for binge eating, even though some had sought it for years [17,38]. It is notable that a few clients did not recognise that they were binge eating and were surprised at the screening outcome. In offering something new (and at times unexpected), GSH was well received and clients expressed gratitude and some sadness that they had not received support sooner. Having the space to talk and feel listened to without judgment in a 1:1 setting was considered essential, even if some of the process was emotionally challenging. MoreLife’s recruitment method for GSH may be useful within other weight management services to capture clients not seeking support for binge eating but consistently struggling with weight management because of their unrecognised eating problem [12]. 

Capturing the perspectives of Guides outside of eating disorder or other mental health services has been recommended previously [39]. The Guides in this study were used to offering structured group weight management interventions. They reflected on their anxieties about facilitating a 1:1 participant-led GSH intervention. However, all reported that they felt it was beneficial to the people they were working with and their anxiety eased as they gained experience and confidence. This reinforces the view that Guides in GSH can come from a variety of backgrounds if they are supported appropriately [24,40].

In terms of the changes made, there was improvement in binge eating in nearly half the clients, evidenced by their scores on the BES. This mirrors the 50% success observed in other low-intensity interventions for eating disorders [24,25]. Even clients who did not show these clear changes, described their eating behaviours as, “*moving in the right direction*”. These findings are consistent with our previous research, showing improvements in binge eating that don’t always reach statistical significance [25,27,28]. Taking a more detailed and qualitative view enables us to see that changes were multifaceted and ongoing. Clients improved in their understanding of their relationship with food and made other positive eating-related changes relevant to binge eating that may not be captured on the BES (such as not shopping when hungry and not keeping food in the bedroom). Follow-up quantitative and qualitative research is required to determine how and whether positive changes in eating continue. Investigations with larger samples may help in triangulating the changes in client’s experiences with routine outcome measures and following this over time.

Difficult emotional experiences are common in people with binge eating problems [3] and improvements for some clients were documented in the interviews. These included decreased shame, guilt, perfectionism, self-criticism, reduced focus on other peoples’ perceptions of them. They were present alongside increased confidence and self-esteem, and feeling more in control of their eating (and better prepared for future weight management support). Correspondingly, most clients demonstrated some (small) improvement across GAD-7, PHQ-9 and SWEMWBS questionnaire scores. The bi-directional relationship between feeling better (mood improvement and well-being) and an improvement in binge eating aligns with the emotional regulation model of binge eating [4]. Binges are associated with challenging emotions and food may be used to try and control these feelings (as per the emotion regulation model). This can lead to short-term relief but an increased likelihood of future difficult emotions resulting in subsequent binges [5]. Thus, if people feel better, they may be less likely to binge, and vice versa. 

During their GSH journey clients described wider lifestyle changes (e.g., increased exercise, volunteering and seeking social support). They related these to feeling more capable, better able to problem solve, and feeling in control of making further changes. This increase in self-efficacy contrasts with reports of low self-efficacy in individuals with BED and obesity, and this as a predictor of post weight management weight gain [41,42]. Future GSH evaluations may consider exploring this further through the addition of a specific measure such as the Self-Efficacy for Self-Help scale to capture these changes [43].

In terms of facilitators of change, clients did not believe they would have made the positive changes, or engaged in the intervention, on their own. The therapeutic relationship was pivotal in motivating clients, improving engagement and facilitating positive changes. This has been demonstrated across several GSH interventions for eating disorders and in other low-intensity programmes for anxiety and depression [23,39,44,45,46,47]. To nurture a positive therapeutic relationship practitioners must view the individual within their context, responding respectfully to individual needs [48]. The ability to personalize and/or tailor GSH was particularly pertinent as it is a transdiagnostic intervention. Some information included in the manual (e.g., relating to managing compensatory behaviours) was not relevant, potentially “*tempting*”, and thus needed to be managed sensitively. This points to the importance of training and supervising Guides to support clients. Finally, part of a positive therapeutic relationship is the collaborative understanding between facilitator and recipient. Guides were clear that in GSH this involved setting expectations for what an intervention can and cannot achieve and what the responsibilities of each party are [49]. Similar findings were reported in work by Traviss-Turner et al. [20] where participants reflected on their naivety as to how much work was required and the level of commitment necessary to engage with GSH effectively. It was the Guides’ role to set realistic expectations from the outset. Expectations influence adherence, treatment satisfaction and outcomes. Unrealistic expectations are associated with an absence of positive psychological change and are an important focus for addressing by Guides [44,50]. 

A second facilitator of positive change was the intervention itself. The combination of a thought-provoking, de-stigmatising, and psychoeducational manual with 1:1 sessions with supportive Guides (as hypothesized) facilitated an increase in self-awareness, self-efficacy, confidence, and the motivation to make positive changes. An increase in client self-awareness was observed in another GSH intervention for binge eating and this was surmised to have facilitated the motivation for clients to continue with the intervention [39]. 

The final facilitator was client-specific namely, clients’ willingness to be open and honest. Key were their ability to accept they had a problem that needed to change and to assume responsibility for making the change. These are fundamental to the success of “self-help”. Similar themes of client motivation and hope have been found within the “common factors” literature [51,52,53]. Due to the active nature of the intervention client factors are considered particularly important in the success of GSH [54]. In this project clients displaying such helpful attributes also described wider help-seeking behaviours, such as engaging in therapy/mindfulness classes, and seeking and/or receiving positive social support. These behaviours indicate a motivation to support themselves and access to the external resources to do so.

In terms of barriers, Guides reflected on difficulties with client attendance and engagement, acknowledging some were, “*complex clients living complex lives*”. This mirrored our earlier pilot study. When recruited, 33 people were eligible, 22 commenced the programme, and 13 completed the twelve weeks and all support meetings [28]. Adherence and retention are challenging across GSH interventions and this warrants further investigation [39]. In particular, how can we develop training for Guides working within weight management settings, and what will help these clients focus on managing their eating disorder? The interviews revealed barriers such as mental and physical ill health, a lack of helpful social support, a focus stuck on weight loss, and external life events (e.g., financial difficulties, changing/losing jobs). Many of these are outside of Guides’/clients’ control even if revealed, but signposting to other sources of support, including emphasizing social support, is possible within GSH interventions. 

One further (and hypothesized) challenge was the persistence of a weight management (loss) goal. GSH for binge eating will not in itself impact on weight. The expectation was that all clients would rejoin the weight management programme after the GSH intervention and would be better able to succeed. However, the desire to lose weight doesn’t diminish during GSH. For those for whom GSH is not appropriate or does not lead to a relief in binge eating, then following a stepped-care model in which more intensive psychological treatments could be offered, such as individual CBT-E, should be explored [19]. In addition, there are likely people who may benefit most from being offered GSH after they have managed some degree of weight loss. How best to sequence interventions for binge eating and weight management and who would benefit from the different sequencing requires deserves further dedicated study.

### 4.1. Strengths

In terms of strengths, we gave a voice to service users with lived experience of obesity. We listened to client experiences of embarking on an intervention to help them manage their binge eating. Second, we captured the experience of staff working in a weight management service who we trained to act as Guides in GSH. Third, the study was sensitive to both the facilitators and barriers to change during the intervention. Fourth, we were able to associate interview themes with changes in the questionnaire outcome measures.

### 4.2. Limitations and Practical Implications

In terms of limitations, the sample size was small, although it was considered adequate for the thematic analysis conducted in the study. We were not able to gain follow-up data on weight outcomes during the weight management intervention, for practical and ethical reasons. There is a need for a larger and longer-term study to help professionals working in weight management to identify people who are experiencing problematic binge eating and properly investigate whether helping people manage their binge eating has benefits to weight management outcomes. Similarly, the sequencing of these interventions is important, as referred to above.

## 5. Conclusions

The themes resulting from these interviews with clients and Guides speak to the perceived value of offering GSH for binge eating before clients embark on weight management. Interventions for binge eating are not generally available in this setting. For some clients it was the first time they had been given space to talk about their binge eating. Others did not recognize that they engaged in binge eating and were not seeking support for it. Having confirmation that there may be a way to address binge eating came as a relief.

GSH offers a focused, programme-led, and supported intervention [24] that clients and Guides agree can improve confidence, wellbeing, and the social lives of people living with obesity. However, it is an intervention that aims to address disordered eating rather than weight management. It is therefore important that Guides help set realistic expectations around outcomes. Weight management services and Guides require training in identifying who may benefit from GSH for binge eating. They also need to be able to ask whether this is the right time for clients to start on what is fundamentally a patient-led intervention. Starting well is especially important in GSH interventions for eating disorders [24]. Are clients in a position to start the intervention now, or what needs to be different so that they can start in the near future? Any training or support of Guides must emphasize the importance of this. Finally, additional research is needed to determine longer-term effectiveness and how best to sequence GSH for binge eating with weight loss interventions.

## Figures and Tables

**Table 1:** Client characteristics and outcome measures.

Client		Gender & Age	Ethnicity	BMI at Referral	BES Scores		PHQ-9 Scores		GAD-7 Scores		SWEMWBS Scores	
					Pre	Post	Pre	Post	Pre	Post	Pre	Post
C-Ant	M	31–40	Multiple ethnic background	46.1	34 (sev)	30 (sev)	11 (mod)	7 (mild)	4 (min)	4 (min)	20 (ave)	22 (ave)
C-Ann	F	21–30	White British	68.0	35 (sev)	17 (no BE)	13 (mod)	11 (mod)	6 (mild)	7 (mild)	26 (ave)	28 (high)
C-Ray	F	41–50	White British	53.0	35 (sev)	30 (sev)	20 (sev)	20 (sev)	18 (sev)	14 (mod)	13 (low)	14 (low)
C-Sue	F	51–60	White British	39.9	37 (sev)	34 (sev)	21 (sev)	20 (sev)	10 (mod)	8 (mild)	16 (low)	16 (low)
C-Mol	F	51–60	White British	39.6	38 (sev)	32 (sev)	19 (mod/sev)	13 (mod)	12 (mod)	11 (mod)	21 (ave)	23 (ave)
C-Ela	F	41–50	Multiple ethnic background	50.7	38 (sev)	33 (sev)	14 (mod)	9 (mild)	3 (min)	0 (min)	23 (ave)	25 (ave)
C-Joy	F	61–70	White British	46.3	36 (sev)	18 (mild mod)	12 (mod)	9 (mild)	8 (mild)	3 (min)	23 (ave)	23 (ave)
C-Bob	M	51–60	White British	42.3	32 (sev)	17 (no BE)	22 (sev)	19 (mod sev)	17 (sev)	13 (mod)	13 (low)	19 (low-ave)
C-Tia	F	61–70	White British	49.2	34 (sev)	5 (no BE)	16 (mod sev)	12 (mod)	10 (mod)	10 (mod)	20 (ave)	23 (ave)

Note. Sev (severe), mod (moderate), min (minimal) are descriptors of the scores on BES, PHQ-9, and GAD-7. High, ave (average), and low are descriptors of scores on the SWEMWBS.

## Data Availability

The data presented in this study are available on request from the corresponding author.
